# The rs78378222 prevalence and the copy loss of the protective allele A in the tumor tissue of diffuse large B-cell lymphoma

**DOI:** 10.7717/peerj.10335

**Published:** 2020-11-12

**Authors:** Elena N. Voropaeva, Yuriy L. Orlov, Tatiana I. Pospelova, Anna A. Gurageva, Mikhail I. Voevoda, Vladimir N. Maksimov, Olga B. Seregina, Maria I. Churkina

**Affiliations:** 1 Research Institute of Internal and Preventive Medicine, Institute of Cytology and Genetics of Siberian Branch of the Russian Academy of Sciences, Novosibirsk, Russia; 2 The Digital Health Institute, I.M. Sechenov First Moscow State Medical University, Moscow, Russia; 3 Novosibirsk State University, Novosibirsk, Russia; 4 Novosibirsk State Medical University of the Ministry of Health of the Russian Federation, Novosibirsk, Russia

**Keywords:** Medical genetics, Nucleotide polymorphism, Diffuse large B-cell lymphoma, TP53 gene, Cancer, Genotyping, rs78378222, Protective allele

## Abstract

**Background:**

Rare single nucleotide polymorphisms (SNPs) are likely to be a crucial genetic factor for human diseases, including cancer. rs78378222 is rare SNP in 3′-untranslated region (UTR) of *TP53* gene leading to disturbance of 3′-end mRNA processing. The frequency of rs78378222 varies in several studied populations. The meta-analysis of 34 genome-wide association studies indicated that rs78378222 was significantly associated with an increased risk of cancer overall. Bioinformatic analysis indicates that somatic loss of the protective A allele of rs78378222 occurs in the tumor tissue of some malignant. The goal of the current study is to document the rs78378222 prevalence and evaluate the copy loss status of the protective allele A in the tumor tissue of patients with diffuse large B-cell lymphoma (DLBCL).

**Methods:**

Total DNA was isolated from FFPE-samples and peripheral blood of patients with DLBCL and comparable in age and sex controls. rs78378222 genotyping was performed by the PCR-RFLP method using restriction endonuclease *Hin*dIII. Direct Sanger’s sequencing was used to confirm the presence of C allele of the rs78378222. The search for *TP53* gene mutations was carried out by Sanger’s direct sequencing method, according to the IARC protocol.

**Results:**

The result of genotyping of 136 DNA samples from DLBCL tumor tissue suggested that frequency of the rs78378222 was 11/136 (8.1%). Rare allele C frequency was 11/272 (4.2%). A total of 5/11 DLBCL rs78378222 heterozygous samples had the heterozygosity loss in the *TP53* gene. Only one of these cases was combined with *TP53* gene mutations which have proven oncogenic potential—p.Arg196Gln, other four cases have not mutations in the coding regions of gene.

**Conclusions:**

At the stages of DLBCL initiation or progression a loss of the protective allele A of rs78378222 occurs. Further efforts are needed to study possible molecular mechanisms underlying somatic alterations in DLBCL in this region of the *TP53* 3′-UTR as well as functional studies to illustrate how the presents of rs78378222 may affect tumor progression of lymphoma.

## Introduction

*TP53* gene (OMIM No. 191117) is located on 17p13.1. The dominant transcript of mRNA has a length 2586 b.p., including eleven exons and 3′- and 5′-untranslated regions (UTRs) (Diederichs et al., 2016). *TP53* gene is a central member in the molecular network of constancy and integrity of the genome, which controls cell cycle regulation and DNA repair. Its activation leads to upregulation of genes involved in the intrinsic (*PUMA*, *BAX*) and extrinsic (*TNFRSF10B*, *FAS*) pathways of apoptosis (Ciardullo et al., 2019). Consequently, *TP53* is considered a key tumor suppressor gene.

Experimental studies on mice models suggested that the restoration of the gene *TP53* function is sufficient for regression of some cancer types. The studies carried out on *TP53*-knockout mice and rats showed that malignant lymphomas are the prevailing form of neoplasms (McCoy et al., 2013).

В-lymphocytes undergoing stress tend to have p53-depended apoptosis, in contrast to other cells that have cell cycle arrest, p53-independent apoptosis or necrosis in stress condition (Wen et al., 2019). Besides, *TP53* gene aberrations in B-lymphocytes leads to less effective functioning of intracellular signaling pathways that can arrest cell cycle in G1 and G2 phases; DNA repair defects; more effective cell adaptation to hypoxia and angiogenesis stimulation; reduction control under telomere structure and differentiation inhibition. For these reasons, the disturbance of programed cell death due to dysfunction p53 is a basis for development and progression of lymphoproliferative disorders (Voropaeva et al., 2017). Moreover, as the *TP53* gene plays an important role in mediation of chemotherapeutic agents’ action and action of target drugs, the deficit of its function leads to formation of multidrug resistance phenotype of lymphoma cells (Voropaeva et al., 2015).

The vast majority of studies dedicated to the role of mutations in nucleotide sequences of *TP53* were focused on analysis of 5–8 gene exons, while the UTRs were ignored. Nevertheless, the changes in these regions may have direct biological effect on B cells maturation and may activate lymphomagenesis (International Cancer Genome Consortium, 2010).

High-throughput sequencing techniques have allowed us to significantly deepen our understanding of the genetic alterations that may be responsible for tumor growth. In addition to the protein-coding sequences, the major non-coding fraction of the genome can be affected by tumor-promoting mutations (Diederichs et al., 2016). Thus, Li et al. (2013) first showed that mutations in UTRs of the *TP53* gene are frequently observed in neoplasms. 5′- and 3′-UTR do not act as a matrix for the protein synthesis, but they are a part of highly conservative elements of the gene. According to their data analyses, the majority of DLBCL patients had the mutations in 3′-UTR in *TP53* and almost all identified replacements lied in the location of confirmed earlier or suggested by in silico analysis micro-RNA binding sites.

Recently hundreds of SNPs in *TP53* have been identified, a part of which is being studied as regard as their influence on DLBCL susceptibility (Voropaeva et al., 2015, 2014, 2016; Pospelova et al., 2010). One of the SNPs of *TP53* gene 3′-UTR (rs78378222) leading to change of normal polyadenylation signal sequence (Ciardullo et al., 2019).

rs78378222 is considered to be affected by the negative natural selection and malignant neoplasms are thought to be the mechanism of such selection (Stacey et al., 2011). It is assumed that this SNP may be a common causal genetic variant for different cancer types in different populations (Zhou, Yuan & Yang, 2012). In whole genome studies rs78378222 was described as a risk factor of prostate cancer, glioma, basal cells carcinoma, esophageal cancer, colorectal adenoma (Wang et al., 2016). The risk-associated allele is a “C,” resulting in an alternative polyadenylation signal (AATACA) replacing the canonical polyadenylation signal (AATAAA) (Li et al., 2013). However, in the literature there are reports of fluctuations in the frequency of detection of rs78378222 in various studied populations: from the absence of detection of this marker to its detection with a frequency of 5–6% (Wang et al., 2016; Rao et al., 2014).

Experiments on cell cultures have shown that the rare C allele compared to the A allele of the rs78378222 leads to both a significant decrease of the p53 protein expression and a decrease of the level of programed cell death induction under the influence of genotoxic factors. (Li et al., 2013).

Bioinformatic analysis by Wang et al. (2015) suggests how the rare variant rs78378222 may affect the function of p53. It has been shown that the С allele break the correct termination of synthesis and processing of the 3′-end of mRNA. It was shown, that the gene transcripts containing the rare allele C of the rs78378222 are on ~3 kb longer than the gene transcripts containing allele A (Wang et al., 2015).

Simultaneously, it was shown that a normal copy of the A allele rs78378222 is lost in the tumor tissue, suggesting a two-stroke theory of carcinogenesis for which both alleles are altered, one at the germline and the second by somatic alteration (Wang et al., 2015).

However, Wang et al. (2015) have shown that loss of the protective common allele A of rs78378222 occurs in glioma but not in lung cancer. Thus, the loss of protective allele A may not be observed in all tumors. Given the presence of such facts the goal of the current study is to document the rs78378222 prevalence and evaluate the copy loss status of the protective allele A in the tumor tissue of patients with one of the most frequent variant of Non-Hodgkin’s lymphomas—diffuse large B-cell lymphoma (DLBCL).

## Materials and Methods

Total DNA was isolated from FFPE-samples of lymph nodes and extranodal tumor lesions biopsies of 136 patients with DLBCL. We used tissue sections in which at least 50% of the cells were tumor cells. Genomic DNA from blood of independent 150 patients with DLBCL and comparable in age and sex 170 controls was isolated by phenol-chloroform extraction method using proteinase K.

**Figure 1:**
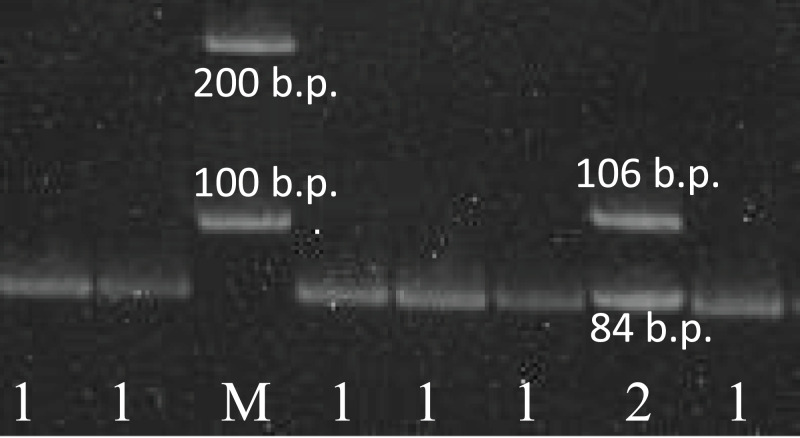
The results of the rs78378222 genotyping. The results of the rs78378222 genotyping by PCR-RFLP method (endonuclease *Hin*dIII). 1–84 b.p. (genotype A/A); 2–106+84 b.p. (genotype A/C); K− negative control; M – 100 b.p. molecular weight marker.

Complete clinical data were available for 280 DLBCL patients: 148 men and 132 women, who were admitted to Novosibirsk Hematological Center during years 2012–2018. As many as 85% of these patients had advanced (III–IV) stages of the disease and more than 60% of them had a poor prognosis according to the International Prognostic Index (IPI). More detailed clinical parameters are presented in the Table 1. The study was approved by the Ethics Committee of the Research Institute of Internal and Preventing Medicine (approval number #2019-01). All patients and controls are ethnical Russians. All study participants provided written informed consent to participate in a research study. The study was carried out in accordance with the World Medical Association (WMA) Declaration of Helsinki (2000) and the Protocol to the Convention on Human Rights and Biomedicine (1999). Sensitivity of the PCR-RFLP and sequencing methods in revealing the presence of unequal amounts of alleles was proven earlier. It was shown that the presence of differences even 10–20% of allele in the total template mixture could be detected by restriction digestion analysis and by sequencing (Siddique et al., 2005). Genotyping of the rs78378222 in *TP53* gene was performed by the PCR-RFLP method, described below. The PCR reaction mixture (25 μl/reaction) contained 75 mM Tris-HCl pH 9.0, 20 mM (NH_4_)_2_SO_4_, 0.01% Tween 20, MgCl2 10 mM 1.5 μl, mixture of deoxynucleotidetriphosphates 10 mM 0.5 μl, 1 u.a. SynTaq DNA-polymerase (Syntol, Moscow, Russia) preparations, 0.1 mkM each of the primers (forward 5′-CACACAGGTGGCAGCAAAGCT-3′ and reverse 5′-AGCACATCTGCATTTTCACCCC-3′) and 1 μl 0.1 mkg/ml genome DNA. The reaction was carried out in thermocycler with using the following conditions: initial denaturation stage—5 min at 95 °C; followed by 31 cycles of denaturation stage—30 s at 95 °C, annealing stage—30 s at 60 °C, extension stage—45 s at 72 °C; post extension stage—3 min. The product of PCR was incubated with 10 u.a. restriction endonucleasae *Hin*dIII in thermostat at 37 °C during 12 h. The fragments of hydrolysis were visualized in 5% polyacrylamide gel. The distributions of bands on the electrophoregram and results interpretation are shown in Fig. 1. The PCR conditions were chosen in such a way as to avoid running out of amplicon in excessive amounts. Restriction endonuclease was added to the test tube in excess and kept for a long time. We believe that these conditions allowed us to avoid the possibility of genotyping error due to undigested amplicons.

**Table 1:** Clinical parameters of the DLBCL patients study group (*n* = 280).

Parameter	Frequency
Age	
<70 years	107 (38.2%)
M 70 years	173 (61.8%)
Sex	
M	148 (52.9%)
F	132 (47.1%)
ECOG score	
0+1	169 (60.4%)
2-3	111 (39.6%)
B-symptoms	
No	113 (40.4%)
Yes	167 (59.6%)
IPI score	
0–2	107 (38.2%)
3–5	173 (61.8%)
Stage	
I–II	42 (15.0%)
III–IV	238 (85.0%)
Extranodal involvement	
No	145 (51.8%)
Yes	135 (48.2%)
Leukemization	
No	199 (71.1%)
Yes	81 (28.9%)
LDH level	
Normal	153 (54.6%)
Elevated	127 (45.4%)

**Note:**

ECOG, Eastern Cooperative Oncology Group; IPI, International Prognostic Index; LDH, Lactate dehydrogenase.

**Figure 2:**
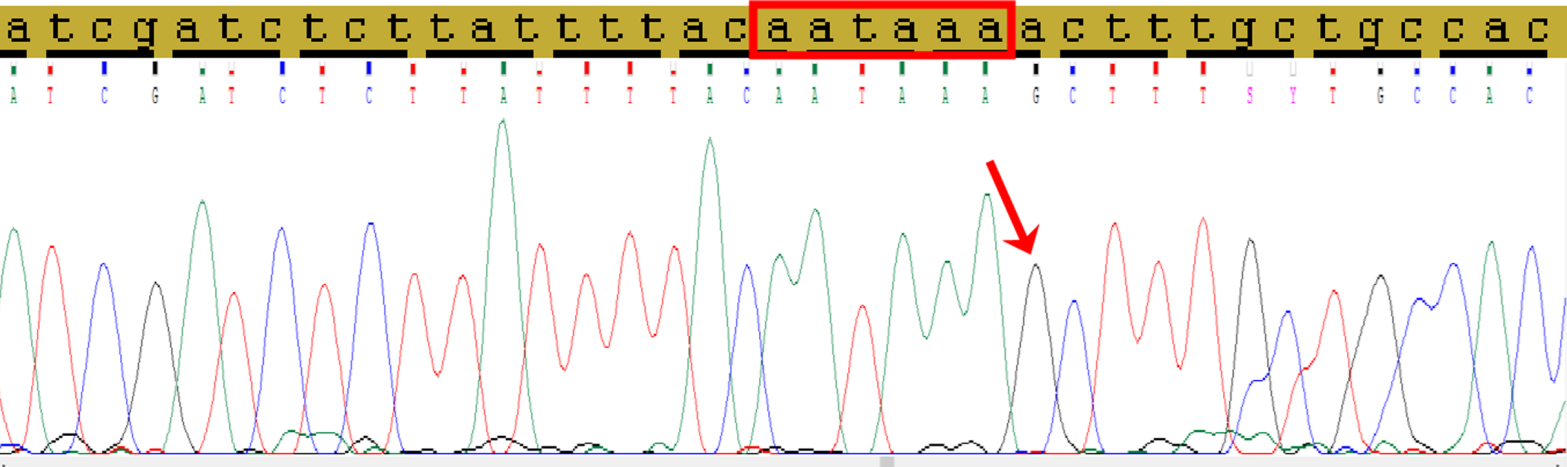
The fragment of 3′-UTR of *TP53* gene containing the rs78378222. The normal polyadenylation signal is highlighted by the red rectangle, the single nucleotide replacement c.*1175A>G as a result of incorporation the site restriction into the reverse primer for RFLP analysis is shown by red arrow.

Direct Sanger’s sequencing was used to confirm the presence of C allele of the rs78378222 (Fig. 2).

Search for *TP53* gene mutations was carried out by Sanger’s direct sequencing method, according to the IARC protocol (http://p53.iarc.fr/download/tp53_directsequencing_iarc.pdf). The PCR products were cleared using microcolumns with SephadexТМ G-50 medium (USA). The sequencing of samples was carried out by the method of capillary electrophoresis on the Hitachi 350 Genetic Analyser (Applied Biosystem, Foster City, CA, USA) using BigDye® Terminator kit v 1.3. and v 1.1. (Applied Biosystems, Foster City, CA, USA) and polymer POP-7. The sequencing results analysis and fragment alignment was conducted by program Chromas, SeqScape v.2.7, Sequence Scanner. The NG_017013.2 sequence of the *TP53* gene was used as a reference.

The comparison of alleles and genotype frequencies of rs78378222 between groups was done by Pearson’s χ^2^ test in 2 × 2 contingency tables. Differences were considered statistically significant at threshold *p* < 0.05.

## Results

The result of genotyping of 136 DNA samples from tumor tissue of patients with DLBCL suggested that genotype A/C of rs78378222 frequency was 11/136 (8.1%). Rare allele C frequency was 11/272 (4.2%). We determined the frequency of polymorphism in healthy tissue patients with lymphoma and controls.

As shown in Table 2, there were no significant differences in distributions of rs78378222 alleles and genotypes between controls and cases of DLBCL (*p* = 0.400) or healthy and tumor tissue (*p* = 0.150) of DLBCL patients. The genotype frequencies of the SNP in both cases and controls were in agreement with the Hardy–Weinberg equilibrium.

**Table 2:** Frequency distributions of rs78378222 alleles and genotypes between DLBCL patients and controls.

rs78378222	Cases *N* (%)		Controls *N* (%)	Cases blood vs. Controls		Cases blood vs. Cases tumor		
	Blood	Tumor		χ^2^	*р*	χ^2^	*р*	
Genotype	А/А	144 (96.0)	125 (91.9)	166 (97.6)	0.71	0.40	2.13	0.15
	А/С	6 (4.0)	11 (8.1)	4 (2.4)				
	С/С	–	–	–				
Allele	А	294 (98.0)	261 (95.8)	336 (98.8)	0.70	0.40	2.07	0.15
	С	6 (2.0)	11 (4.2)	4 (1.2)				

rs78378222 minor allele frequency in healthy controls was higher than the in the “1000 genomes project” (13/5008 (0.3%), *p* = 0.004) (Abecasis et al., 2012), which can be explained by the different prevalence of the allele in populations of different ethnicities. Thus there were no significant differences in rs78378222 minor allele frequency between current study controls and Estonian (*p* = 0.151) (https://www.ncbi.nlm.nih.gov/snp/rs78378222) and Northern Sweden (*p* = 0.440) (https://www.ncbi.nlm.nih.gov/snp/rs78378222) cohorts (shown in Table 3).

**Table 3:** rs78378222 minor allele frequency in several studies.

Cohort	*N* (%)	p*	References
Current study	4/340 (1.2)	–	–
1000 genomes	13/5008 (0.3%)	0.004	Abecasis et al. (2012)
GnomAD	373/31380 (1.2)	1.0	Karczewski et al. (2019)
Estonian	107/4480 (2.4)	0.151	(https://www.ncbi.nlm.nih.gov/snp/rs78378222)
Northern Sweden	11/600 (2.0)	0.440	(https://www.ncbi.nlm.nih.gov/snp/rs78378222)

**Note:**

* *p*-value vs. current study.

According to design of the rs78378222 genotyping by PCR-RFLP analysis, the presence of rare allele C destroys the site of restriction endonuclease *Hin*dIII. It is known that fixing the tissue by formalin and long-term storage of samples in paraffin blocks may results to artificial DNA changes. For this reason we carried out Sanger’s direct sequencing of 3′-UTR fragment of *TP53* gene, containing the rs78378222 from DNA samples of patients with DLBCL who have minor allele C.

**Figure 3:**
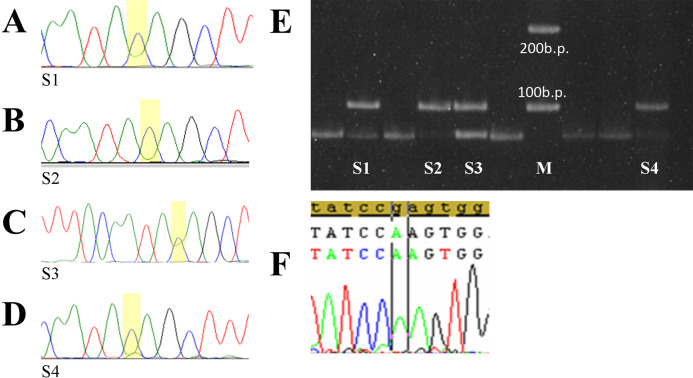
Selected tumor samples with rs783782. Selected tumor samples with rs78378222: (A–D) sequences chromatograms; (E) results of genotyping by PCR-RFLP method; (F) p.R196Q homo-zygote mutation. S1, 2, 4—tumor samples with C/C genotype rs78378222 (loss of heterozygosity in the TP53), S3—sample with A/C genotype rs78378222, M – 100 b.p. molecular weight marker.

Five of tumor samples had prepotency С allele “dose” on electrophoregrams and sequences chromatograms (Figs. 3A and 3B). Sensitivity of the PCR-RFLP and sequencing methods in revealing the presence of differences even 10–20% of allele in the total template mixture was proven earlier (Siddique et al., 2005). Since the cases of rare homozygous genotype C/C rs78378222 detection in normal tissue have not been described previously, and DNA extraction was carried out from paraffin blocks contained not less than 50% of tumor tissue, the gained results demonstrated a heterozygosity loss in the *TP53* of the rs78378222 in tumor tissue of patients DLBCL.

Only one of these cases was combined with *TP53* gene mutations which can be considered pathogenic—p.Arg196Gln (Fig. 3C). Codon 196 is phylogenetically conservative. It is a part of the beta-sheets of DNA-binding domain and one of the “hotspots” of the *TP53* gene mutations (Bouaoun et al., 2016). Mutation p.Arg196Gln is described in the COSMIC database (Genomic Mutation ID COSV52674826) and the IARC *TP53* mutation database (MUT_ID2461) for various tumors (Bouaoun et al., 2016; https://cancer.sanger.ac.uk/cosmic/mutation/overview?id=96071916#references). Analysis with using tools which predicts possible impact of an amino acid substitution on the structure and function of a human protein PolyPhen-2 (Polymorphism Phenotyping v2) and SIFT shows that p.Arg196Gln refers to the number of damaging substitutions. Functional assessment of p53 mutants revealed that this mutation leads to loss of transactivation activity of protein (Marutani et al., 1999; Monti et al., 2003).

In other four cases rs78378222 with heterozygosity loss were the only aberration in *TP53*. Detailed description of the mutations identified in the Russian DLBCL patient’s cohort was described in previous publication (Voropaeva et al., 2017).

## Discussion

Currently, the international research projects such as Cancer Genome Atlas Research Network (TCGA) and International Cancer Genome Consortium (ICGC) have shown the presence in tumors a large number of driver mutations in the regions of genes that do not encode the protein sequence (International Cancer Genome Consortium, 2010; Esgueva et al., 2012). Aberrations in 3′-UTR of *TP53* gene may be a universal carcinogenesis mechanism, playing role in pathogenesis of DLBCL as well. In particular mutations in 3′-UTR of *TP53* gene may alter the interactions with regulatory miRNAs or cause the destruction of conservative sequence of polyadenylation signal and the occurrence of additional signals (Diederichs et al., 2016; Li et al., 2013). Li et al. (2013) first reported the prognostic value of aberrations in 3′-UTR of *TP53* gene in DLBCL patients.

Rare variants are more likely to have a functional impact and tend to have a greater effect size than do common variants. Thus, rare variants are likely to be a crucial genetic factor for human diseases, including cancer (Gorlov, Gorlova & Frazier, 2011; Gorlov, Gorlova & Sunyaev, 2008).

Enciso-Mora et al. (2013) in the whole genome sequencing study described rs78378222. It is rare SNP in 3′-UTR of *TP53* gene leading to disturbance of 3′-end mRNA processing. This polymorphism change canonical sequence of *TP53* gene polyadenylation signal. This signal is required for the detection of sequences by polyadenylation complex, cleavage, polyadenylation and export of mature RNA to cytoplasm (Li et al., 2013).

Recently, several studies have investigated the association between a newly reported rs78378222 and cancer risk, but generated inconsistent findings. The meta-analysis on the basis of high-grade data from 34 Genome-wide association studies indicated that rs78378222 was significantly associated with an increased risk of overall cancer. Furthermore, stratified analyses indicated that rs78378222 increased the risk of cancers: nervous system, skin and others (Wang et al., 2016). Among millions of polymorphisms in human genome the rs78378222 has the strongest correlation with basal cell carcinoma (Diskin et al., 2014). rs78378222 is considered as one of the mechanism of cancer predisposition in Li-Fraumeni syndrome (LFS) and its variant, Li-Fraumeni-like Syndrome (LFL), autosomal dominant disorders that are characterized by predisposition to multiple early onset cancers (Macedo et al., 2016).

There was no purpose to estimate the association of polymorphism with a predisposition to lymphoma. The power of current study is not enough to resolve this purpose. Identification the role of polymorphism in predisposition to development DLBCL requires a multi-center joint study, what can be the basis for our research in the future.

The goal of the current study is to document the rs78378222 prevalence and evaluate the copy loss status of the protective allele A in the tumor tissue of patients with Diffuse large B-cell lymphoma (DLBCL). It is suggested that with at least some malignant neoplasms, the loss of the protective allele A in the tumor may occur. Thus Wang et al. have shown that loss of the allele A occurs in glioblastoma, but not in lung cancer (Wang et al., 2015).

The result of genotyping of 136 DNA samples from tumor tissue of patients with DLBCL suggested that frequency of the rs78378222 was 11/136 (8.1%). Rare allele C frequency was 11/272 (4.2%). And it was higher than the rs78378222 minor allele frequency in the “1000 genomes project” (*p* < 0.001), which could be explained by both the different prevalence of the allele in populations of different ethnicities and tumor origin of the samples.

The frequency of rs78378222 varies in different cancer patients studies: from the absence of detection of this SNP in oral, cervical and breast cancer patients from South India to its detection with the frequency 5.4% in cohort LFS/LFL patients (Wang et al., 2016; Rao et al., 2014). The detailed analysis Genome Aggregation Database (GnomAD) (Karczewski et al., 2019) showed that rs78378222 allele C frequency in European population is 1.7% whereas in Latino and African—is 0.4% and 0.2%, in East Asian and Ashkenazi Jewish—is 0%.

Since data on the rs78378222 prevalence in the Russian population is not presented in the literature and specialized databases, we defined the frequency of polymorphism in healthy tissue patients with lymphoma and controls. There were no significant differences in distributions of rs78378222 alleles and genotypes between controls and cases of DLBCL or healthy and tumor tissue DLBCL patients. There were no significant differences in rs78378222 minor allele frequency between current study controls and Estonian (https://www.ncbi.nlm.nih.gov/snp/rs78378222) and Northern Sweden cohorts (shown in Table 2) (https://www.ncbi.nlm.nih.gov/snp/rs78378222).

Remarkably, none of the DNA samples from healthy tissue from cases and controls has rs78378222 in homozygous state. However, the part (5/11) of analyzed DLBCL samples had the heterozygosity loss in *TP53* gene in carriers of genotype A/C of the rs78378222. One of these cases was combined with p.Arg196Gln *TP53* gene mutation which have proven oncogenic potential—, other four cases have not mutations in the coding regions of gene.

Attention to this SNP is due to the fact that rs78378222 is thought to be the unique SNP of *TP53* gene with the reduced p53 function. The *TP53* mRNA level decrease in the cells having the rs78378222 compared to the cells without this polymorphism but with another closely-spaced marker rs114831472 was confirmed. Allele C of rs78378222, as compared with the A allele, dramatically lowers p53 protein expression and cellular apoptosis (Li et al., 2013; Macedo et al., 2016). It is also suggested that since the micro-RNA binding site is located on 3′-UTR of gene, SNP of 3’-UTR *TP53* including rs78378222 may affect the gene expression by canceling, weakening or creation of new binding sites (Gorlov, Gorlova & Sunyaev, 2008; Macedo et al., 2016).

Findings another study suggest that allele C rs78378222 lead to haploinsufficiency of p53. It is situation in which the total level of a gene product produced by the cell is about half of the normal level and that is not sufficient to permit the cell to function normally (Gorlov, Gorlova & Sunyaev, 2008).

Thus, rs78378222 presents a rare instance of *TP53* alleles with unambiguous reduced function, in contrast with vast majority of coding sequence mutants which are associated with loss of р53 function, dominant negative effect of mutants and/or gain of uncharacteristic for wild-type p53 functions (Li et al., 2013).

Research group Wang et al. (2015) performed the integrated analysis of TCGA data and suggested a functional mechanism for germline rare variant in *TP53* rs78378222. Using RNA sequencing data, they observed aberrant transcripts with ~3 kb longer than normal for those individuals. Using exome sequencing data, they further showed that loss of haplotype carrying common protective allele A occurred somatically in tumor tissue. Thus analysis suggests rare risk allele C disrupts mRNA termination, and an allelic loss of a genomic region harboring common protective allele A occurs during initiation or progression some tumors, for example glioblastoma (Wang et al., 2015).

## Conclusions

At the stages of DLBCL initiation or progression a loss of the protective allele A of rs78378222 occurs. Further efforts are needed to study possible molecular mechanisms underlying somatic alterations in DLBCL in this region of the *TP53* 3′-UTR as well as functional studies to illustrate how the presents of rs78378222 may affect tumor progression of lymphoma.

## Supplemental Information

10.7717/peerj.10335/supp-1 Supplemental Information 1 Genotyping data for rs78378222 in 280 patients. Anonymised clinical data for 280 patients including sex, age, symptoms, stage, and rs78378222 genotyping.

10.7717/peerj.10335/supp-2 Supplemental Information 2 Raw genotyping data on rs78378222 including sequencer screenshots. Nucleotides variants in rs78378222 for the patients under study resent raw data for the analysis.

## Additional Information and Declarations

### Data Availability

The following information was supplied regarding data availability: The raw measurements are available in Tables 1–3. Anonymized patient information is available in a Supplemental File.
